# Implications of Lung Compliance Phenotypes in COVID-19-Induced Acute Respiratory Distress Syndrome

**DOI:** 10.7759/cureus.105397

**Published:** 2026-03-17

**Authors:** Harish Gidda, Tsz Hin Ng, Susan Szpunar, Ashish Bhargava, Rene Franco

**Affiliations:** 1 Pulmonary and Critical Care Medicine, Henry Ford St. John Hospital, Detroit, USA; 2 Pharmacy, Henry Ford St. John Hospital, Detroit, USA; 3 Medical Education, Henry Ford St. John Hospital, Detroit, USA; 4 Infectious Disease, Henry Ford St. John Hospital, Detroit, USA

**Keywords:** covid 19, covid induced ards, lung compliance, mechanical vent, pulmonary critical care

## Abstract

Background: Early literature suggested a phenomenon where coronavirus disease 19 (COVID-19)-induced acute respiratory distress syndrome (ARDS) presented with high respiratory system compliance (RC) in some patients. Thus, it posed a challenge to conventional ARDS ventilator management strategies.

Research question: Are there different phenotypes of lung compliance among mechanically ventilated patients with COVID-19-induced ARDS? Does the RC phenotype impact the clinical outcome?

Study design and methods: This was a single-center historical cohort study that included patients who were mechanically ventilated for COVID-19-induced ARDS and who met the Berlin Criteria for ARDS. RC was calculated at intubation, for eight weeks, and at extubation. Data on demographic factors and comorbidities were also collected.

Measurements: RC was calculated by tidal volume/pressure. Descriptive statistics, chi-squared test, Student’s t-test, and multivariable logistic regression were used to analyze the data.

Main results: Compliance groups were derived from the collected data. Most patients (73.6% to 89.4%) had severely reduced (≤40 ml/cmH₂O) static RC, and (10.7% to 26.4%) had RC ≥40 ml/cmH₂O. At week one and at extubation, there was a statistically significant difference in mortality between high versus moderate versus low RC groups: week one: (57.1% versus 66.7% versus 87.7%, respectively, p = 0.02); extubation: (83.3% versus 58.8% versus 87.2%, p = 0.01, respectively). Remdesivir administration, older age, and higher Charlson Comorbidity index scores were associated with increased mortality (4.2 OR 95% CI [1.61, 10.87]), (1.03 OR 95% CI [1.003, 1.07]), and (1.31 OR 95% CI [1.03, 1.67]), respectively. Declining PaO2/FiO2 (P/F) ratios were associated with lower RC.

Interpretation: COVID-19 ARDS presents a range of RC and different phenotypes, which may require different ventilator strategies. Given the increased mortality at extubation in patients with low RC, it poses the possibility of a bedside prognostication tool. Older age, number of comorbidities, and Remdesivir use were associated with increased mortality. Lastly, declining P/F ratios were associated with lower RC.

## Introduction

Acute respiratory distress syndrome (ARDS) is a devastating complication of coronavirus disease 2019 (COVID-19) infection. Traditionally, in ARDS, respiratory system compliance (RC) is decreased because of diffuse alveolar damage and edema resulting from increased permeability. Other patient characteristics, such as age, hypertension, diabetes, chronic kidney disease (CKD), and obesity, may also affect respiratory system compliance and have a potential role in ventilator management in COVID-19 ARDS [1,2]. Most studies note that decreasing lung compliance in COVID-19 ARDS is associated with higher mortality rates, similar to traditional ARDS, but some reports are to the contrary [1-4]. Furthermore, many studies report that COVID-19 ARDS behaves similarly to non-COVID-19 ARDS [5-7] and would likely benefit from lung-protective ventilation with low tidal volume and higher positive end-expiratory pressure (PEEP) [1-3,8,9].

There are reports suggesting that COVID-19 ARDS presents with different respiratory mechanics compared to traditional ARDS [10,11]. This has implications for the ventilator management strategy for COVID-19 ARDS.  The disagreement on how COVID-19 affects respiratory system compliance and ventilator management poses an intriguing area of study. There is literature suggesting that there are two different types of COVID-19 ARDS [12,13]. In these studies, Type 1 COVID-19 ARDS occurs when the compliance is normal to high, and these patients have minimal alveolar recruitability. Additionally, overdistension may occur with higher PEEP, causing vasoconstriction and worsening gas exchange via ventilation/perfusion mismatch. Type 2 COVID-19 ARDS has characteristics similar to traditional ARDS, with lower respiratory compliance and improvement with lung-protective ventilation strategies [12]. In non-COVID-19 ARDS, lung compliance is inversely proportional to mortality [14]. In this study, we sought to observe differences in RC in patients with COVID-19-induced ARDS who were on invasive ventilator support to determine if there were differing compliance noted in COVID-19 ARDS and, if so, could possibly suggest alternative ventilator strategies. Furthermore, we assessed the implication of RC on mortality.

This manuscript was presented at the CHEST National Conference 2023 [29].

## Materials and methods

Research questions   

What is the association between respiratory system compliance and outcomes in COVID-19 ARDS, and are there high and low respiratory compliance phenotypes in COVID-19 ARDS? Are there risk factors or patient characteristics that can identify a respiratory system compliance phenotype for COVID-19 ARDS?

Hypothesis statements   

H01: There is no association between respiratory system compliance and COVID-19 ARDS outcomes. HA1: There is an association between respiratory system compliance and COVID-19 ARDS outcomes. 

Study design and methods 

This was a historical cohort study done via retrospective chart review. Subjects included patients aged 18 or older. Admitted to the intensive care unit (ICU) at Ascension St. John Hospital in Detroit between March 2020 and March 2021, who tested positive for COVID-19 or were diagnosed at an outside facility. Patients needed to meet the ARDS criteria, defined by the Berlin criteria, with a PaO₂/FiO₂ (P/F) ratio of less than 300, bilateral infiltrates, and non-cardiogenic pulmonary edema [15]. Only patients who were intubated and mechanically ventilated during their admission were included. Patients who did not meet ARDS criteria were excluded. Patients were further divided into three subgroups based on their respiratory system compliance. Severely reduced/low RC, ≤40 ml/cmH₂O; moderately reduced RC between 40-50 ml/cmH₂O; and mild reduction/high RC ≥ 50 ml/cmH₂O. The cutoffs for respiratory system compliance were data-driven, with typical normal respiratory system compliance ranging from 50 to 100 ml/cmH₂O. As this was a retrospective study, ventilator management was up to the discretion of the physician caring for the patient at the time. We calculated static respiratory system compliance (Cstat) by the equation Vt/(Pplat-PEEP) [16,17], and dynamic respiratory system compliance (Cdyn) was calculated by Vt/(Ppeak-PEEP) (Vt = tidal volume, Pplat = plateau pressure, Ppeak = peak pressure). For patients ventilated with airway pressure release ventilation (APRV), the RC was calculated by Vt/((P High × T High) + (P Low × T Low)/(T High + T Low)) (P High = highest pressure used, P Low = lowest pressure used, T Low = time at low pressure, T High = time at high pressure) [18]. These measurements were taken at the time of intubation, then on a weekly basis for up to eight weeks and on the day of extubation. Data were collected on demographic factors, clinical factors, and the comorbidities used to compute the Charlson Comorbidity Index. Data were analyzed using the chi-squared test, Student’s t-test, and multiple logistic regression. All data were analyzed using IBM Corp. Released 2025. IBM SPSS Statistics for Windows, Version 29. Armonk, NY: IBM Corp., and a p-value less than 0.05 was taken to indicate statistical significance. All data was handled in compliance with HIPAA. This project was approved by the Ascension Health IRB RMI20220253. 

Power analysis 

Puah et al. [9] studied the association between lung compliance phenotypes and mortality in COVID-19 patients with acute respiratory distress syndrome.  They found that mortality was higher in patients with high compliance than in patients with low compliance, 8 (33.3%) vs. 5 (11.6%), respectively. To find this difference with an alpha error rate of 0.05 and 90% power, we needed to include 81 patients with low compliance and 81 patients with high compliance, for a total of 162 patients.  With 162 patients, we appeared to be underpowered, so we increased the sample size to 256 patients (based on the number of patients who fit the inclusion and exclusion criteria out of a sample of 500 charts). 

Statistical analysis  

Descriptive statistics were calculated to characterize the study groups.  Continuous variables were described as the mean with standard deviation or the median with range.  Categorical variables were described as frequency distributions.  Univariate analysis was done using Student’s t-test and chi-squared analysis.  For non-normally distributed data, the Mann-Whitney U test and Kruskal-Wallis test were employed.  Multivariable analysis was done using logistic regression.  All data were analyzed using IBM Corp. Released 2025. IBM SPSS Statistics for Windows, Version 29. Armonk, NY: IBM Corp., and a p-value of 0.05 or less was considered to indicate statistical significance.

## Results

Medical records of 256 patients were examined; comorbidities and patient demographics can be seen in Table 1. The mean age of the study group was 63.4 ± 13.4 years; 56.5% (144) of the study group were male. Of note, 54.1% (138) of the population was Black/African American. Sepsis, hypertension, and diabetes were the comorbidities with the highest prevalence in this study population, 97.3%, 70.2%, and 38%, respectively. 

**Table 1:** Describing the overall characteristics of the study group, demographics, and comorbid conditions COPD: Chronic Obstructive Pulmonary Disease, CKD: Chronic Kidney Disease, TIA: Transient Ischemic Attack, DM: Diabetes Mellitus

Category	Number	Total	Percent
Sepsis	248	255	97.3
Hypertension	179	255	70.2
COPD	53	255	20.8
Asthma	20	255	7.8
Smoking History	53	255	20.8
Pulmonary embolism	13	255	5.1
Diabetes	116	255	45.5
Heart Failure	51	254	20.1
DM Without Complication	97	255	38
DM With Complication	20	255	7.8
Peptic Ulcer Disease	7	255	2.7
Hemi/Paraplegia	3	255	1.2
Any Malignancy	28	255	11
Solid Tumor	14	255	5.5
Severe CKD	83	255	32.5
Mild Liver Injury	88	255	34.5
Moderate Liver Injury	47	255	18.4
Severe Liver Injury	36	255	14.1
Myocardial Infarction	36	255	14.1
Stroke or TIA	30	255	11.8
Dementia	20	255	7.8
Peripheral Vascular Disease	18	254	7.1
Connective Tissue Disease	7	255	2.7

**Figure 1:**
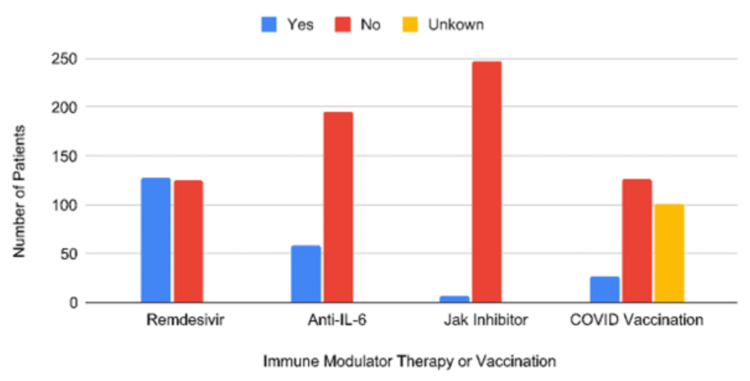
Number of patients who received immune-modulating therapy or COVID-19 vaccination

The frequencies of immunomodulatory therapy can be seen in Figure 1. Notably, 50.4% of patients had received remdesivir therapy. Only 23.1% of patients had received anti-interleukin-6 (IL-6) therapy. Very few patients, 2.7%, received Janus kinase inhibitor (JAK) therapy. Vaccination rates showed that only 10.6% had received at least one dose of the COVID-19 vaccination. Remdesivir administration during hospitalization was associated with increased mortality (4.2 OR 95% CI [1.61, 10.87]).

**Figure 2:**
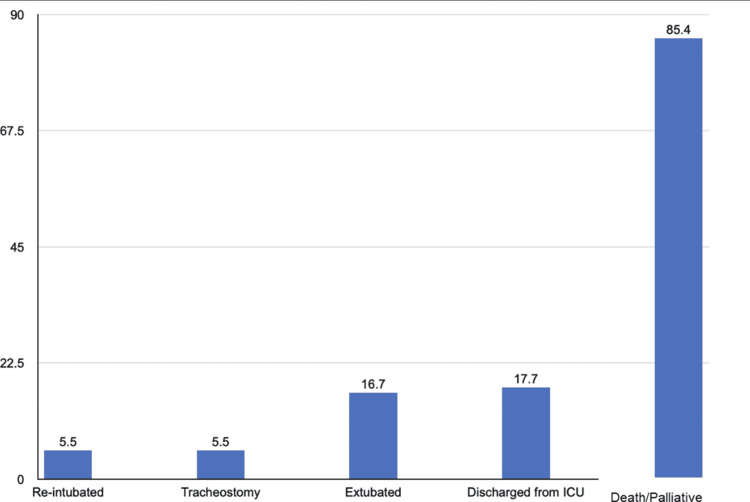
Percentage of patients that were in each primary endpoint group of successful extubation, discharged from the ICU, re-intubated, and dead or palliatively extubated. Percentages seen on the Y-axis and endpoints on the X-axis

Out of 253 patients with available data, only 42 (16.6%) were successfully extubated, with 9 (21.4%) of these patients being re-intubated. A total of 45 patients (17.6%) were discharged from the ICU. Overall, 14 (5.5%) patients received a tracheostomy. The case fatality rate was 85.5%; these results can be seen in Figure 2. 

**Figure 3:**
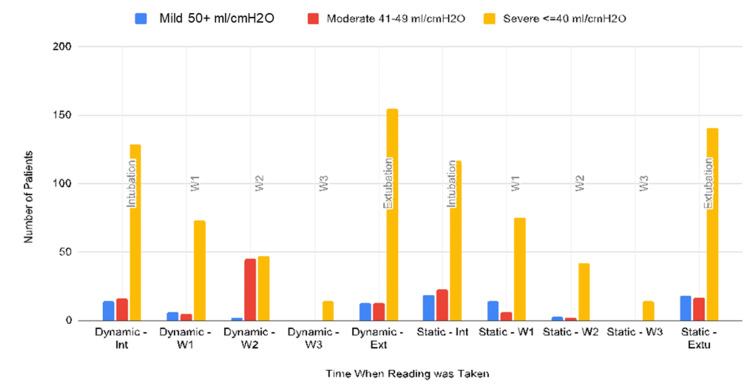
Frequency of patients with mild, moderate, and severe reduction in dynamic and static lung compliance at intubation, extubation, weeks 1, 2, and 3, including re-intubation W: week; Ext: extubation; Int: Intubation Dynamic: Dynamic respiratory system compliance, Static: Static respiratory system compliance, Blue bars: Mild reduction in lung compliance 50+ ml/cmH_2_O, Red bars: Moderate reduction in lung compliance 41-49 ml/cmH_2_O, Yellow bars: Severe reduction in lung compliance </=40 ml/cmH_2_O

Dynamic respiratory system compliance (Cdyn) at the time of intubation showed 14 patients (5.5%) with Cdyn 50+ ml/cmH₂O, 16 patients (6.3%) with Cdyn between 40 and 49 ml/cmH₂O, and 129 patients (50.6%) had Cdyn less than 40 ml/cmH₂O; 96 patients (37.6%) did not have documentation of dynamic lung compliance at intubation. Nineteen patients (7.5%) had a static respiratory system compliance (Cstat) of 50+ ml/cmH₂O at intubation, 23 patients (14.5%) had a Cstat between 40-49 ml/cmH₂O, and 117 patients (45.9%) had a Cstat less than 40 ml/cmH₂O; 59 patients (62.4%) did not have documented static lung compliance at intubation. At week one, mild, moderate, and severe reduction in dynamic lung compliance (Cdyn) was observed in six (2.4%), five (2%), and 74 (28.6%) patients, respectively. A mild, moderate, and severe reduction in Cstat was observed in 14 (5.5%), eight (2.4%), and 65 (25.5%) patients, respectively. One hundred seventy-one patients did not have a documented Cdyn, and 170 patients did not have a Cstat at week one. At week two, a reduction in Cdyn was mild in two patients (0.8%) and severe in 45 patients (17.6%). Two hundred and eight patients did not have Cdyn at week two. Cstat reduction at week two was mild in three patients (1.2%), moderate in two (0.8%), and severe in 42 (16.5%) patients. At week three, only 14 patients had documented static and dynamic respiratory compliance, all with values less than 40 ml/cmH₂O (5.5%). At extubation, Cdyn showed mild reduction in 13 patients (5.1%), moderate reduction in 13 patients (5.1%), and severe reduction in 155 patients (60.8%); 74 did not have a documented Cdyn at extubation. Cstat at extubation showed mild reduction in 18 patients (7.1%), moderate reduction in 17 patients (6.7%), and severe reduction in 141 patients (55.3%); 79 did not have static lung compliance documented. There was not enough data for lung compliance assessments from week four to week eight. Most patients had severely reduced lung compliance at intubation, week one, week two, week three, and at the time of extubation (Figure 3).

**Figure 4:**
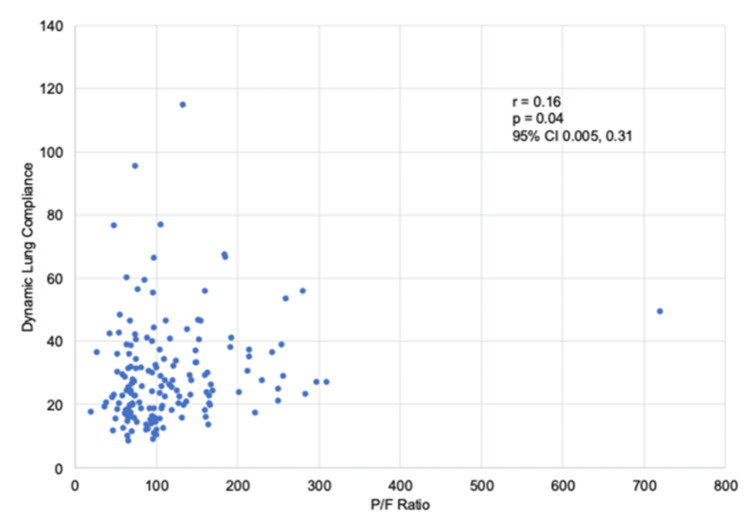
Scattered plot showing correlation of P/F ratio/severity of ARDS to dynamic respiratory system compliance

The majority of patients had severely reduced P/F ratios. When comparing P/F ratios to lung compliance, there was a mild correlation between declining P/F ratios and the respiratory system compliance correlation coefficient (r) = 0.16, with p-value = 0.04 (Figure 4). The data suggest that patients with severely reduced P/F ratios can present with a wide range of respiratory system compliance, but are more likely to present with declining respiratory system compliance (Figure 4).

Patients who underwent death/palliative extubation had a mean age of 64.6 ± 12.9 years compared to 56.2 ± 14.5 years for those who survived (p < 0.001). For patients who survived, the mean time on the ventilator was 14.8 ± 14.0 days compared to 9.3 ± 8.8 days for those who expired (p = 0.001). Of the 37 patients who did not undergo death/palliative extubation, the mean modified Charlson Comorbidity Index was 2.5 ± 2.5 versus the 218 patients that did expire, 3.4 ± 2.5 (p = 0.06). 

**Figure 5:**
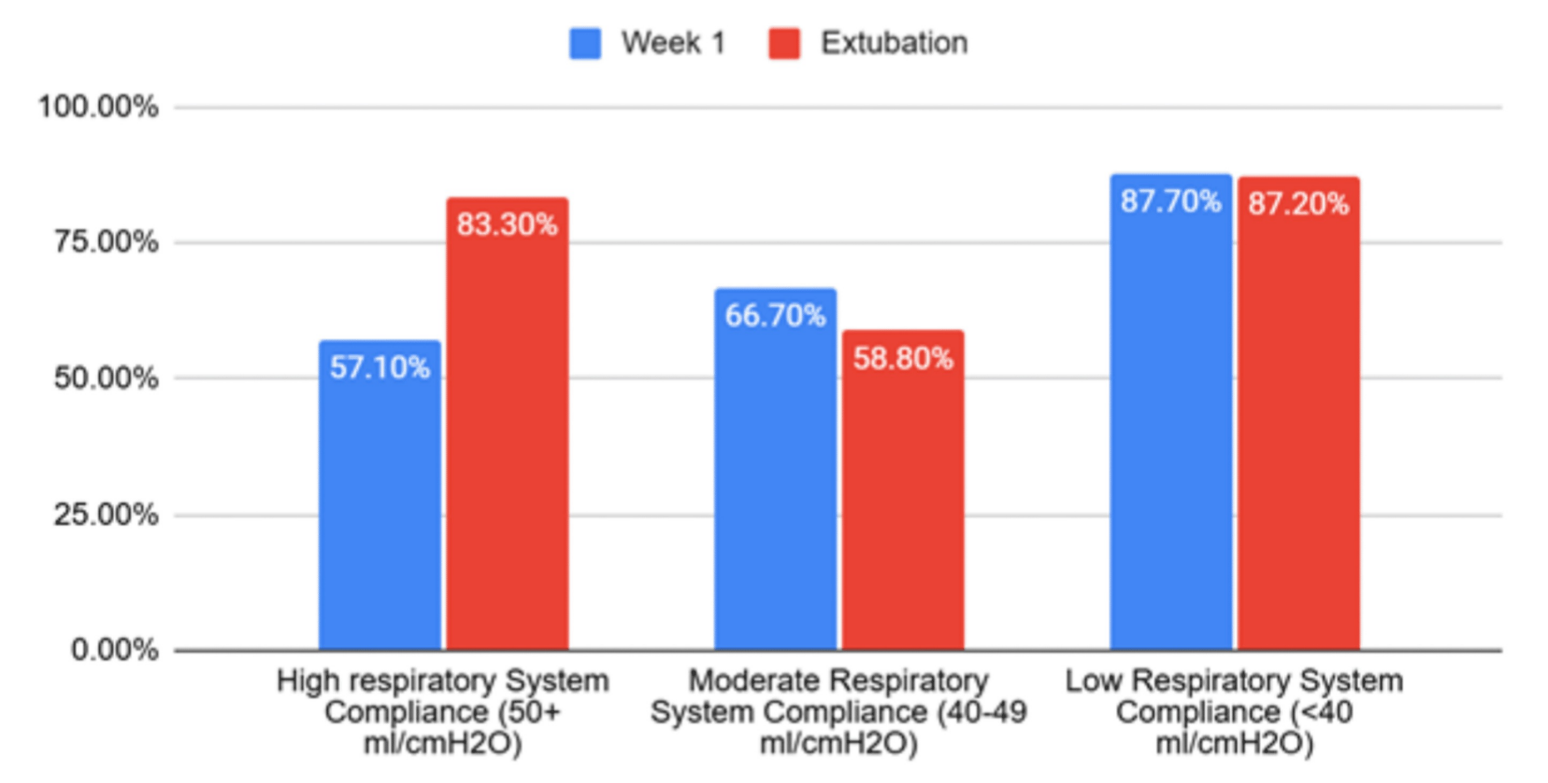
Chi-squared analysis showing the percentage of patients with high, moderate, and low respiratory system compliance at week 1 and extubation

Cdyn did not show a statistically significant difference in outcomes of mortality among different lung compliance groups. A higher mortality rate was in the groups with more severe Cstat reduction at week one: mild reduction 57.1%, moderate reduction 66.7%, and severe reduction 87.7% (p=0.02). There was no statistically significant difference in mortality at weeks two and three between groups. At the time of extubation, a difference in mortality was observed in the different Cstat groups: mild reduction 83.3%, moderate reduction 58.8%, and severe reduction 87.2% (p=0.01) (Figure 5).

From multivariable analysis, every one-year increase in age increased the risk of death by 3.4% (OR=1.034, 95% CI 1.003, 1.07, p=0.03); use of remdesivir increased the risk of death 4.2 times (OR=4.2, 95% CI 1.61, 10.9, p=0.003); and every one-unit increase in Charlson score increased the risk of death by 31% (OR=1.31, 95% CI 1.03, 1.7, p=0.03). 

## Discussion

The main objective of this study was to identify distinct respiratory system compliance groups among patients who underwent mechanical ventilation for COVID-19 ARDS. Additionally, we aimed to explore the implications of these phenotypic differences. Our study revealed a wide range of lung compliance values, with most of the patients (73.6% to 89.4%) falling into the severely reduced RC group (≤40 ml/cmH₂O). A smaller subset experienced mild to moderate reductions in RC (10.7% to 26.4%) with values ≥40 ml/cmH₂O, suggesting the existence of multiple phenotypes of COVID-19 ARDS. Compliance groups were derived from the data collected. Additionally, the data showed a wide range of RC for severely reduced P/F ratios, but with a trend towards low P/F ratios correlating with low RC. To note, patients who were re-intubated were not analyzed separately. This suggests the possibility of more than one COVID-19 ARDS phenotype. Due to the high mortality of this study, there was minimal data beyond week three of intubation, which may have affected these results. 

Our study findings indicate that older patients and those with higher scores on the Charlson comorbidity index exhibited a higher mortality rate. This observation aligns with previous research conducted by others [18-24]. Interestingly, we observed a 4.2-fold increased risk of mortality among patients who received remdesivir. It is worth noting that the National Institute of Health guidelines recommend remdesivir for patients with mild to moderate COVID-19 infections who are at risk of disease progression but do not require mechanical ventilation [21]. We hypothesize that patients in this study may have received remdesivir too late in the disease course or possibly after intubation as a salvage therapy, as they were the sickest patients in the hospital. Additionally, patients may have been intubated late in their disease course and may have received remdesivir during escalating oxygenation and ventilation requirements prior to intubation, as the study only looked at patients who were intubated and mechanically ventilated.

In our cohort, most patients experienced severely reduced RC. We hypothesize that this is likely explained by later intubation of COVID patients at our institution, leading to patients with more advanced COVID-19 ARDS being intubated and therefore included in this study. This is further supported by the higher mortality seen in our study (85.5%) when compared to other studies (60% [23], 35% [25]). Although still debated, differences in respiratory mechanics between COVID-19 ARDS and non-COVID-19 ARDS have been repeatedly reported [25,26], specifically higher compliance in early COVID-19 ARDS [27,28]. 

​​We also investigated the significance of these RC groups in relation to mortality, which we previously defined as death or palliative extubation. Patients with severely reduced static lung compliance (≤40 ml/cmH₂O) at week one post-intubation and at the time of extubation exhibited increased mortality. Our findings suggest that low respiratory system compliance in COVID-19-induced ARDS may be linked to increased mortality compared to high lung compliance phenotypes. This correlates with other causes of ARDS, where lower lung compliance is associated with increased mortality [9]. Additionally, we saw a correlation between lower P/F ratios, indicating worsening ARDS, and decreasing respiratory system compliance. However, within this data set, it is seen that for a given P/F ratio, there was a range of compliance, which can be reflected in the correlation coefficient (r) = 0.16 with a p-value of 0.04 (95% CI 0.004, 31) (Figure 4). Our study may be underpowered to fully evaluate the potential of multiple phenotypes for a given P/F ratio. Further investigations will be needed to elucidate the presence of multiple COVID-19 ARDS compliance phenotypes and optimal ventilator strategies for high RC phenotypes versus low RC phenotypes. Where traditional lung-protective strategies with high PEEP may not be optimal.

Retrospective review of data from a single medical center is a limitation of this study. An important limitation of our study is that few patients had data points beyond week two, which may have affected the longitudinal analysis of compliance in COVID-19 ARDS. This may have been attributed to the high mortality rates seen in this study population at week one. Higher mortality was observed in patients with severely reduced RC, and a large proportion of patients had a severe reduction in RC at the time of intubation (Cdyn 50.6% and Cstat 45.9%). Additionally, the severity of the disease, duration of the disease prior to intubation, modalities and duration of non-invasive ventilator support prior to intubation, and sample size could have influenced the validity of these findings. The overall mortality in this cohort is higher than that reported in other COVID-19 cohorts [23,24], which may limit the generalizability of our findings. 

## Conclusions

COVID-19-induced ARDS may present with a range of RC phenotypes. In this study, most patients who were intubated and mechanically ventilated for COVID-19 ARDS exhibited a severe reduction in respiratory system compliance. Patients in this study also had high mortality. Additionally, patients in this study were intubated later in the disease course, which may have been the reason for lower respiratory system compliance and may explain the association of remdesivir and increased mortality. As patients may have received remdesivir as salvage treatment or prior to intubation, which was likely later in their disease course. Moreover, patients with a severe reduction in RC at week one and at the time of extubation had increased mortality. Higher mortality seen in patients with reduced Cstat at the time of extubation may provide useful information when making decisions about mechanical ventilation liberation, but further studies would be needed to delineate its utility. Although our study suggests there may be a possibility of different compliance phenotypes of COVID-19-induced ARDS, the true prevalence should be confirmed in larger cohort studies. Additional investigation into optimal ventilation strategies for different phenotypes is an area of opportunity for future research.

In summary, this study suggests a possibility of high and low respiratory compliance phenotypes, and lower respiratory system compliance may be associated with higher mortality. Further studies would be needed to truly elucidate if there are multiple COVID-19 ARDS phenotypes. Further studies are also needed to determine if differing ventilator strategies would be impactful with high versus low respiratory system compliance and to determine the implications of respiratory system compliance in COVID-19 ARDS.
